# A systematic review of stereotactic radiosurgery for metastatic spinal sarcomas

**DOI:** 10.1007/s11060-024-04892-z

**Published:** 2024-11-28

**Authors:** Trent Kite, Stephen Jaffe, Vineetha Yadlapalli, Rhea Verma, Jenna Li, Stephen Karlovits, Rodney E. Wegner, Matthew J. Shepard

**Affiliations:** 1 Department of Neurosurgery, Allegheny Health Network Neuroscience Institute, Pittsburgh, PA USA; 2 Drexel University College of Medicine, Philadelphia, PA USA; 3 Division of Radiation Oncology, Allegheny Health Network Cancer Institute, Pittsburgh, PA USA; 4 Allegheny Singer Research Institute, Allegheny Health Network, Pittsburgh, PA USA

**Keywords:** SRS, Sarcoma, Metastases, Spinal oncology

## Abstract

**Purpose:**

Sarcomas metastasizing to the spine are a rare entity. Ideally an En-bloc resection is necessary to achieve durable local control (LC) rates. However, anatomical constraints often limit the degree of tumor resection. Because of this, other therapeutic modalities either replacing or as an adjuvant to resection are necessary. Stereotactic radiosurgery (SRS) is a reasonable candidate therapy.

**Methods:**

We conducted a systematic review of the literature using the following databases: PubMed, Science Direct, and Cochrane library. We used a combination of the following terms connected by boolean operators: “Metastatic Sarcoma, Sarcoma of the Spine, Spine Sarcoma, Metastasis, stereotactic radiosurgery, SRS.” All retrospective and prospective cohorts, as well as randomized control trials reporting on patients with histopathologically confirmed metastatic sarcomas of the bony elements of the vertebrae, thecal sac, cord, or associated soft tissues of the spine were included. We excluded animal studies, case reports, case series, patients < 18 (pediatric cohorts), review articles and meta-analyses. No date filters were applied to our search.

**Results:**

Our final analysis included 5 studies ranging from 2009 to 2024 reporting on 260 patients and 371 associated lesions. Leiomyosarcoma was the most frequently reported histologic subtype (60%). Most lesions were localized to the thoracic spine (48.6%). 75% of studies reported a median dose < 30 Gy, and achieved biologically equivalent doses (BEDs) ranging from < 50–100. Pooled 1-year median survival was 64.5% (IQR: 61.8–75.10). Pooled 1-year median LC was 86% (IQR: 79.4–88.5). Three of five studies (60%) for OS and 4/5 (80%) for LC had data availability suitable for meta-analysis. The 1-year OS and LC rates proportions across these studies were 67% (proportion = 0.67, 95% CI: 0.57–0.75, p = 0.07, I^2^ = 63%), and 84% (proportion = 0.84, 95% CI: 0.78–0.89, p = 0.10, I^2^ = 52%) respectively. Median follow up across all studies was 18 months (IQR:12.7–31.3).

**Conclusions:**

SRS is a reasonable alternative therapy in either the up front, salvage or adjuvant setting which can facilitate durable LC.

**Supplementary Information:**

The online version contains supplementary material available at 10.1007/s11060-024-04892-z.

## Introduction

Sarcomas are a rare and heterogenous group of cancers with a predilection towards metastasis [1–5]. A large proportion of patients undergoing primary radiotherapy or surgical resection for their index lesion will experience distant metastasis [1, 6, 7]. Lung represents the greatest site of metastasis while the spine comprises a smaller proportion of the overall metastatic burden [4, 5]. Nonetheless, when sarcomatous lesions metastasize to the spine, they are responsible for tremendous morbidity and mortality [4, 5, 8]. Sarcomatous spinal metastases can present with severe functional limitation and pain, posing a therapeutic challenge [1]. An aggressive approach to tumor eradication must be balanced against preservation of function and quality of life [4, 5]. Contemporary management of sarcomatous spinal metastases has become increasingly multimodal consisting of radiotherapy, surgical resection, and chemotherapy [1, 9, 10].

The primary goal of management is to achieve adequate local control (LC) rates [1]. Complete resection of the metastatic lesions is correlated with improved survival, and therefore preferred if possible if the systemic disease burden is limited [1]. However, complete surgical resection can be limited given the proximity of the spinal cord and other anatomical constraints of the vertebral column [1]. Alternatively, radiotherapy is relatively less limited by anatomical constraints compared to surgical approaches [1].

Historically, conventional external beam radiation therapy (EBRT) was the predominant radiosurgical approach, however this approach has become increasingly displaced by stereotactic radiosurgery (SRS) [1]. SRS is favorable given its increased precision targeting with minimal off target tissue toxicity compared to EBRT, allowing for the maximization of LC with marginal treatment related adverse events [1, 6]. Additionally, EBRT has not demonstrated sufficient evidence for long term LC [11–14].

SRS has previously been demonstrated as efficacious in metastatic spinal lesions [1, 15, 16]. Existing literature has reported LC rates for all metstatic spinal lesions consistently at or above 85% at one year [4, 5, 17–22]. However, little is understood regarding SRS in the setting of sarcomatous histologies [1]. Sarcomatous metastases of the spine are rare and have traditionally been viewed as radioresistant, limiting thier widespread investiagtion in the literature [1, 11, 23–25]. Furthermore, the existing data has a tendency towards heterogeneity in the reporting of thier results [4, 5]. However preliminary data suggest the potential for durable LC rates with limited toxicity, particularly with respect to vertebral compression fractures (VCFs) [1, 12, 25–27].

Given the potential for an improvement in outcomes over traditional surgical approaches we sought to summarize the limited evidence for SRS in sarcomatous metastases of the spine. Additionally, a pooled analysis of selected endpoints will provide a broader perspective on the trends across available literature in this specific tumor subtype.

## Methods

### Search strategy

A Systematic review was performed in accordance with the preferred reporting of items in systematic review and meta-analysis (PRISMA) guidelines [28]. The following electronic databases were utilized in our search: PubMed, Science Direct, and Cochrane Library. Variations of the phrase “((Stereotactic radiosurgery) OR (SRS) OR (Linear Accelerator) OR (LINAC) OR (Stereotaxy)) AND ((Spinal Sarcoma) OR (Spine Sarcoma) OR (Metastatic Sarcoma of the spine) OR (Secondary spine sarcoma))” were searched. No date restrictions were applied as filters in our search criteria. Searches were restricted to clinical trials, randomized controlled trials, and retrospective, prospective or observational cohorts. Two authors (V.Y, T.K) conducted the search independently. Any discrepancies were resolved by a third author (R.V). Our search results spanned 1966 to September 2024.

## Eligibility and exclusion criteria

English articles with the following characteristics were included: patients ≥ 18 years of age with histopathological diagnosis of a sarcoma localized to the bony elements of the vertebral column, thecal sac, cord, or soft tissue elements of the vertebral column, who underwent any type of SRS (linear accelerator (LINAC), stereotactic body radiotherapy (SBRT), Image guided SRS (IG-SRS). We excluded: letter to the editor, case reports, conference abstracts, animal studies, meta-analyses, reviews, editorials, as well articles that included both primary and metastatic spinal sarcoma patients but lacked sub-group analyses sufficient for exclusive extraction of the metastatic lesion data.

Data was extracted independently by two separate authors (T.K, V.Y). The following variables were extracted from eligible studies: demographic and clinical features (number of patients indication for SRS, spinal levels affected, extraspinal disease status etc.). Radiosurgical parameters (median dose, number of fractions, etc.). Finally, information on outcomes (overall survival (OS), progression free survival (PFS), adverse radiation events (AREs) etc.) were collected. All data were collected in a central excel database.

## Quality assessment

The final list of selected articles underwent a round of screening using a specific Joanna Briggs Institute (JBI) checklist designed for cohort studies. Screening was conducted by two separate authors (T. K and V. Y). Articles with scores > 6 were considered good quality for inclusion in the final analysis.5/5 (100%) scored at or above this threshold.

## Statistics

Descriptive statistics were performed using GraphPad Prism (V.10.3.1). Local control Wald 95% confidence intervals (CI) were calculated for studies that reported local control/failure event rates at 1-year (Folkert, Bishop, Shanker, and Kim). Overall survival Wald 95% CIs were also calculated for overall survival for studies that reported event rates at 1-year (Folkert, Bishop, and Kim). Forest plots for 1-year LC and 1-year OS were constructed for 4 and 3 studies respectively. The pooled point estimate reported in these plots represents the proportion of lesions exhibiting local control or survival along with a 95% confidence interval. In both cases the data available to construct point estimates and associated 95% confidence intervals was not available in all 5 studies selected for final review. Therefore, those studies were omitted from the Forest plots. Furthermore, there was insufficient data available in all 5 studies to construct point estimates and associated 95% confidence intervals for OS and LC at 2- and 3-years. Therefore, these analyses were omitted. In order to estimate heterogeneity across the studies included in the analysis we reported an I^2^statistic. Values of < 50%, 50–75%, and > 75% represented low, moderate, and substantial heterogeneity respectively. Neither of the I^2^statistics calculated for LC or OS was greater than 75% and therefore did not require further subgroup analysis.

## Results

### Search results

**Fig. 1:**
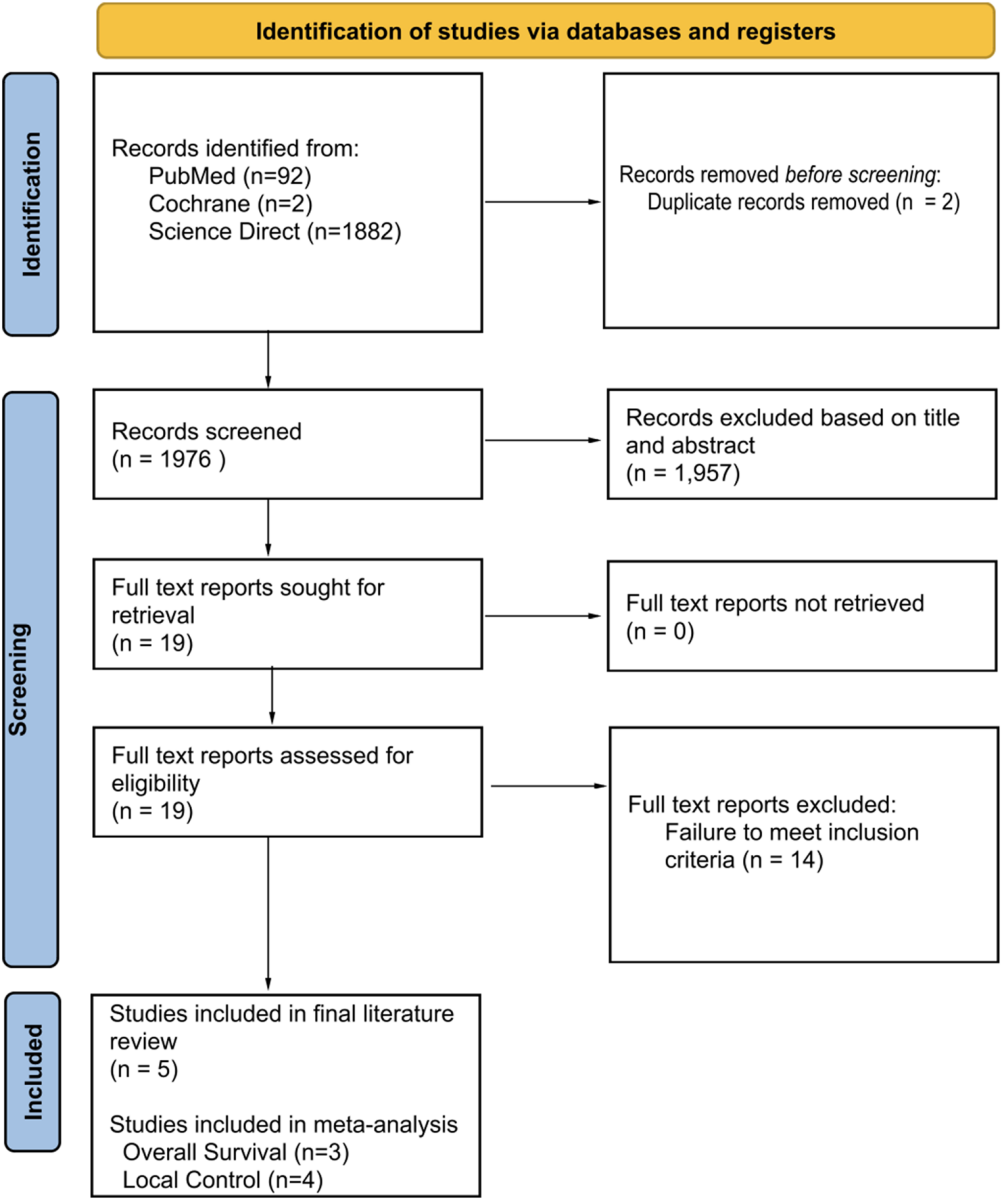
PRISMA flow chart. PRISMA flow diagram detailing the article selection process

After an initial database search, 1,976 articles were identified, and titles/abstracts were screened for relevance and fulfillment of inclusion criteria. After abstract/title screening 19 articles were selected for full text screening. After full text review 5 articles were selected for inclusion in the final analysis. Our selection process is outlined in Fig. 1.

## Baseline lesion characteristics

5 total studies published from 2009 to 2024 were included in the review. These studies accounted for 260 patients with 371 lesions. 287 (77%) lesions underwent SRS for definitive therapy, while 84 (23%) lesions underwent SRS for salvage or adjuvant treatment. 4/5 (80%) studies reported which patients underwent single versus fractionated SRS accounting for 355/371 (95%) lesions. Across these 4 studies, 211/355 (59%) underwent single fraction SRS and 144/355 (41%) underwent hypofractionated SRS. A summary of basic study characteristics is provided in Table 1.

**Table 1:** Baseline study characteristics. Baseline characteristics of the studies included in the review

Study	Date	Number of Patients	Number of Lesions	Indication (N)	Dosing Scheme
Shanker et al. [26]	2024	70	100	Definitive (100)	Single (100)
Kim et al. [1]	2023	44	69	Definitive (51) Salvage (18)	Single (12) Fractionated (53)
Bishop et al. [4]	2015	48	66	Definitive (44) Salvage (22)	Single (31) Fractionated (35)
Folkert et al. [11]	2014	88	120	Definitive (80) Salvage (40)	Single (68) Fractionated (52)
Levine et al. [25]	2009	10	16	Definitive (12) Salvage (4)	NR

Abbreviations: NR: not reported

## Tumor characteristics

Two (40%) studies reported Osteosarcoma as the most frequent histologic tumor subtype. Three (60%) studies reported Leiomyosarcoma as the most common histologic subtype. The Distibution of metastatic lesions along the spine was reported in 3/5 (60%) studies which accounted for 271/371 (73%) lesions. From most frequent to least frequent, lesion distribution across the vertebral levels was as follows: 139 (48.6%) thoracic, 54 (18.9%) lumbar, 38 (13.3%) cervical, 25 (8.7%) sacral, 18 (6.3%) lumbosacral, 6 (2.1%) cervicothoracic, and 6 (2.1%) lumbosacral. Two out of five (40%) studies quantified the proportion of lesions associated with active disease outside of the spine (extraspinal) at the time of SRS. This accounted for 220/371 (59%) lesions. Across the two reporting studies 107/220 (89%) of lesions were associated with extraspinal disease at the time of SRS. A summary table of the specific lesion details is presented in (Supplemental Table 1).

### Radiosurgical parameters

3 (25%) studies used a median dose less than 30 Gy (range: 16–45). 2/3 (67%) studies reporting data on median number of fractions used a median of 3 fractions (range: 2–6). 3/3 (100%) studies reported using a percent PTV coverage equal to or greater than 93%. BEDs across the three reporting studies ranged from < 50–100. A full summary of radiosurgical parameters can be found in Table 2.

**Table 2:** Radiosurgical parameters. Summary of radiosurgical parameters utilized in studied included in the review

Study	Median Dose (Gy)	Median Number of Fractions	PTV Coverage	Biologically Equivalent Dose (BED)
Shanker et al. [26]	24 (16–24)	1	NR	81.6 (81.6–81.6) *
Kim et al. [1]	33 (18–45)	NR	98.8% (93.6-100%) *	100 (60–80) *
Bishop et al. [4]	NR	3 (3–6)	NR	< 50 (11) 50–59 (40) ≥ 60 (15)
Folkert et al. [11]	Single Fx: 24 (18–24) Multi Fx: 28.5 (24–36)	3 (2–6)	Single Fx (93%) Multi Fx (95%)	NR
Levine et al. [25]	30 (20–30) *	NR	98.3% (± 1.2%) **	NR

Abbreviations: Fx: fractions, NR: not reported, PTV: planned target volume

Data reported as median (range) unless otherwise noted

*Reported as median (IQR)

**Reported as a mean value

### Clinical outcomes

**Fig. 2:**
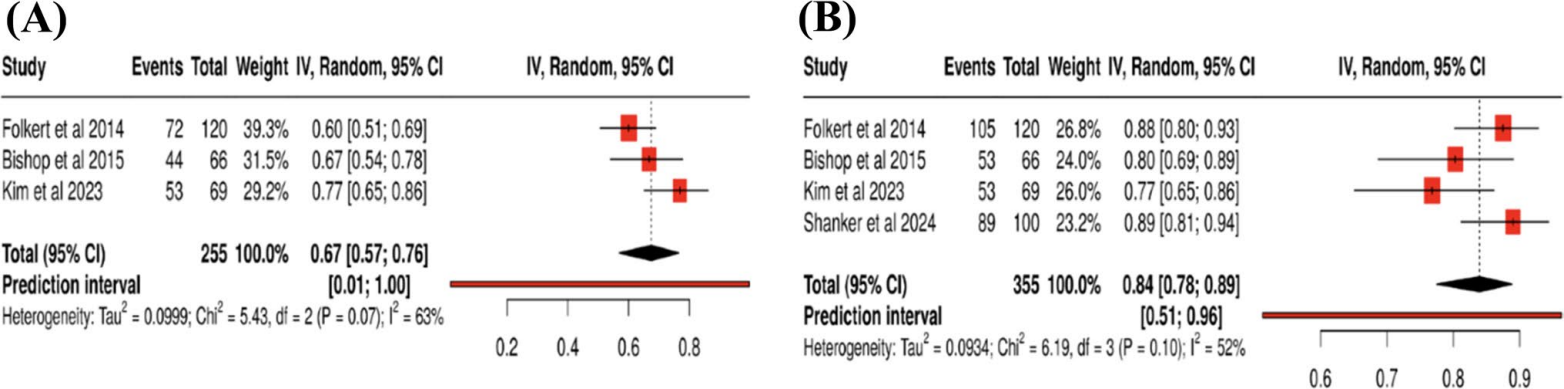
Forest plots demonstrating 1-year LC and OS proportions. (**a**) Proportion of individuals surviving at 1-year of clinical follow up. (**b**) Proportion of individuals exhibiting LC at 1-year of radiographic follow up

Overall, actuarial 1-year OS across 3/5 studies was 67% (Fig. 1a). was reported by 4/5 (80%) studies at a median rate of 64.5 (IQR: 61.8–75.1). 2-year OS rates were reported by 1/5 (20%) studies at 45.9%. 3-year OS rates were reported by 1/5 (20%) studies with a median of 34.3 months (IQR: 26.0-42.7). Figure 2a demonstrates the proportion of OS at 1-year of clinical follow up. The overall pooled actuarial OS across the studies included in the plot was 67% (proportion = 0.67, 95% CI: 0.57–0.75,*p* = 0.07, I^2^ = 63%).

Overall, actuarial 1-year LC across 4/5 studies was 83% (Fig. 1b). Two-year LC rates were reported by 1/5 (20%) studied at 62.9%. Three-year LC rates were reported by 2/5 (40%) studies at a median of 57.7% (54.4–61.0). Figure 2b demonstrates the proportion of LC 1-year of radiographic follow up. The overall pooled actuarial LC across the studies included in the plot was 84% (proportion = 0.84, 95% CI: 0.78–0.89,*p* = 0.10, I^2^ = 52%).

Median follow up time across all studies was 18 months (IQR: 12.7–31.3). 3/5 (60%) studies reported VCF frequency for a median frequency of 4% (IQR: 2.3-9.0). A summary of clinical outcomes is provided in Table 3.

**Table 3:** Clinical outcomes. A summary of the survival, local control, and vertebral compression fracture outcomes in each study

Study	1/2/3-year Overall Survival	1/2/3-year Local Control	Toxicity	Median Follow up Time
Shanker et al. [26]	NR	89.0%	VCF: 9.0%	13 months (IQR:7–25)
Kim et al. [1]	77.8%/54.9%/ 42.7%	77.8%/62.9%/ 54.4%	NR	18.2 months (range: 2.4-153.7)
Bishop et al. [4]	67.0%/NR/ 26.0%	81.0%/NR/ 61.0%	VCF: 4.0%	19 months (range 1-121)
Folkert et al. [11]	60.6%	87.9%	VCF:2 2.3%	12.3 months (range: 1-80.7)
Levine et al. [25]	62.1%/NR/NR	85.9%/NR/NR	VCF: 0%	43.5 months *

Abbreviations: NR: not reported, VCF: vertebral compression fracture

*Reported as a mean value

## Discussion

Overall, we demonstrated that many metastatic sarcomatous lesions of the spine being treated with SRS are receiving SRS as “definitive” therapy (primary treatment). The proportion of patients receiving single versus multifraction therapy is comparable. Most commonly, Leiomyosarcoma is the predominant histology across all articles included in our analysis. The most frequent spinal segment involved is the thoracic spine, while the thoracolumbar segment is least frequently involved. Extraspinal disease status at time of SRS ranged from 35 to 72%. Most of the studies used a median of 3 fractions, PTV coverage ≥ 90%, with a wide range of BEDs (< 50–100). Overall median 1-year survival, and 1 year LC was 64.5% (IQR: 61.8–75.1) and 86% (IQR: 79.4–88.5) respectively. Median follow up across all studies was 18 months (IQR:12.7–31.3). Additionally results from the meta-analyzed data were consistent with these estimates at 67% and 84% for OS and LC at 1-year respectively.

### Radiation parameters

Previously, it has been determined that conventional radiation doses > 60 Gy are necessary to achieve LC for metastatic sarcomatous spinal lesions [4, 5, 27, 29]. However, the incidence of spinal cord toxicity rises steeply with radiation dose [4, 5, 27]. Furthermore, studies examining the role of conventional EBRT in metastatic spinal lesions primarily investigate mixed histologic populations, limiting comparison with SRS treated sarcomatous lesion subgroup analyses [25]. Therefore, interpretation of available SRS literature against EBRT literature in this patient population is difficult. One strategy to overcome this limitation is through the investigation and reporting of biologically equivalent doses (BEDs) in the SRS literature.

Kim et al. were able to achieve median BEDs of 100 (IQR: 60–180) with a median SRS dose of 33 Gy (range 18–45) [1]. Bishop et al. achieved BEDs of > 48 with a dose of 24 Gy per fraction and demonstrated on univariate analysis that BED > 48 Gy was associated with improved LC (81%, HR: 0.33,*P* = 0.04) [4]. Estimation of the BEDs from the median dose prescribed in the cohort of Folkert et al. reveals that single fraction dosing was able to achieve BEDs > 139.2 Gy compared to multifraction dosing reaching BEDs of 82.7 Gy which translated into a survival benefit for those undergoing single fraction SRS compared to multifraction SRS. Furthermore, Shanker et al. established a relationship between the minimum gross tumor volume dose and LC rates [26]. On univariate analysis the authors showed that at a minimum dose of 12 Gy there was a reduced chance of local failure (HR = 0.871, 95% CI: 0.782–0.97, *P* = 0.009) [26]. While it is presently unclear if a reliable dose response relationship exists between SRS and metastatic spinal sarcomatous pathology with respect to LC, there may exist a reliable working dose range within which good LC rates can be achieved [1, 4, 11, 25, 26].

Additionally, there has been debate surrounding the use of single versus multifraction radiation dosing with a general perspective that single fraction SRS has been superior in the setting of sarcomatous metastatic lesions of the spine [1, 4, 11, 25, 26].In the largest SRS study on this tumor population Folkert et al. demonstrated in a multivariate analysis that the use of single fraction SRS at a median single fraction dose of 24 Gy (range: 18–24) compared to multifraction SRS at a median dose per fraction of 28.5 Gy (range: 24–36) was associated with improved LC (HR: 0.35, *P* = 0.03) [4, 11]. Assessing this relationship is difficult as present literature lacks many double arm retrospectives or prospective designs enabling direct comparison. The study by Folkert et al. is the only paper in our review conducting such an analysis. When comparing across studies, Shanker et al. exclusively used a single fraction SRS approach and produced the highest 1-year local control rates at 89% with a median single fraction dose of 24 Gy, similar to Folkert’s cohort [11, 26].

### Local control

One of the most essential elements in managing sarcomatous metastasis to the spine is achieving excellent LC. Identifying and understanding the features which influence tumoral response to SRS is paramount. For example, the extent of vertebral involvement has been demonstrated to limit LC [1]. In the study by Kim et al. the authors reported a significant association between the number of vertebral levels (which corresponded to the extent of metastatic dissemination) treated and LC rates, with a trend towards improved LC rates with less vertebral levels treated (1-year LC for single level: 80.9% versus 2–3 levels: 60.0%) [1]. This finding was not recapitulated in either Shanker of Bishop’s cohorts [4, 26]. This highlights the challenges in target volume delineation and planned target volume coverage (PTV %) against the motivation to decrease adjacent healthy tissue toxicity which is increasingly difficult with increased three-dimensional lesion volume.

Another contemporary debate surrounds the nature of tumor histology and the potential that certain sarcomatous lesions have an inherently greater degree of radioresistance translating to diminished LC. In two studies directly examining this relationship both Kim and Folkert showed no significant relationship with tumor histology and LC rates [1, 11]. Consistent with these findings no relationship between LC and tumor histology was delineated in Bishop et al. [4]. However, Shanker et al. did demonstrate a relationship between time to local failure and lesion histology [26]. In this study Leiomyosarcoma and Ewing Sarcoma histologies exhibited significantly longer times to local failure with respective mean times of 18.6 months and 34.3 months [26]. There is a great deal of diversity in the histological subtypes of sarcomas, implying some degree of therapeutic response heterogeneity. To date no definitive relationship between histologic subtype and response to SRS has been established across multiple studies [1, 4, 11, 25, 26]. In the era of genomics one approach to determining the potential for radioresistance is through large scale genomics. Past research has attempted to use genetic signatures to calculate a radioresistance index across the sarcoma tumor subtypes [1, 30–33]. While the approach is consistent with generalized attempts across all oncology research to quantify tumor behavior in the context of genetic behaviors, there has been a lack of demonstrated clinical utility [1].

Additionally, the treatment setting may have an influence on LC rates. Bishop et al. demonstrated that a patient receiving SRS in the post-operative setting was associated with diminished 1 year LC rates (71% versus 87%) in the definitive group [4]. The authors in this study argue this relationship may be multifactorial and attributable to surgical seeding, higher proportion of patients receiving multifraction regimens versus single fraction in the post-operative setting, and the application of lower radiation doses on average [4]. No significant relationship was reported between prior radiation therapy or surgical resection with the use of single fraction SRS in Folker’s cohort [11]. Future work should emphasize the stratification of patients based on dosing parameters in the post-surgical SRS setting.

Finally, A few studies conducted a detailed examination of patterns of local failure. One such study by Bishop et al. exhibited the most frequent failure type was a marginal recurrence occurring in 78% of local failures [4]. Another study by Shanker et al. revealed a 12% marginal recurrence, which represented the most frequent pattern of local failure similar to Bishop et al. [4, 26]. In contrast, an extended examination of patterns of failure in the Folkert cohort, conducted by Leeman et al. demonstrated a tendency for distant versus local recurrence [24]. In this study the authors demonstrated that only 5.5% of lesions experienced isolated local recurrence, and that 60% of local recurrences had disease occurring at or beyond 3 vertebrae away from the initial treatment volume [24]. A more complete understanding of the patterns of disease progression will facilitate treatment planning with respect to optimal target volume and target volume coverage. If additional evidence consistently demonstrates vertebral involvement distant from the original lesion, then considering additional adjacent vertebrae beyond the target lesion in the original plan may be necessary. However, as previously discussed the addition of more vertebral levels to the SRS plan does not reliably translate into improved LC.

### Overall survival

Overall survival in this patient population remains poor, underscoring the need for improved systemic therapy. In this setting the primary treatment goals for spinal irradiation have historically emphasized LC while minimzing treatment toxicity [1, 4, 11, 25, 26]. One factor which has been reproduced across two studies is the role of extraspinal or total metastatic disease burden on OS [1, 26]. Both studies have exhibited a trend toward decreased survival with increased metastatic disease burden, particularly in the setting of concurrent spinal and extraspinal visceral metastases [1, 26]. Kim et al. reported a decreased OS in patients with solitary metastatic lesions versus spinal lesions and visceral metastases (HR: 5.618, 95% CI: 2.3–13.7) [1]. While Shanker et al. demonstrated a similar trend it was more modest (HR: 1.583, 95% CI: 1.1–2.3, *P* = 0.01) [26]. While overall disease burden is likely to limit outcomes in any metastatic diseased state, it may be a valuable prognostic indicator. Those patients presenting with widespread disease may benefit from a palliative approach compared to an SRS approach designed to achieve aggressive local control and the associated risk of quality-of-life detriment in the context of an unclear survival benefit. Furthermore, it may be important in future studies to determine the level of extraspinal disease control in conjunction with the binary reporting of “present” versus “not present.” Stratifying patients with extraspinal disease on the basis of disease control may reveal a new trend in OS outcomes.

### Toxicity

Given that the overall survival is low in this patient population, consistent with survival in all metastatic diseases, maximizing tumor control, while minimizing toxicity to promote quality of life is a primary consideration. Most frequently the risk of vertebral compression fracture limits the application of radiation to the spine [1, 4, 11, 25, 34–37]. Kim et al. reported a post-SRS vertebral compression fracture frequency of 6.8% in thier cohort [1] Similarly, Bishop et al. reported a vertebral compression fracture frequency of 6% [4]. Shanker et al. reported a low rate of vertebral compression fracture at 9% of thier cohort [26]. In Levine et al., no VCFs were reported [25]. Meta-analytic data derived from Faruqi et al. reporting on the risk of vertebral compression fracture in 2,911 spinal segments reported a raw fracture rate of 13.9% [26, 36]. Similarly, the CCTG SC24/TROG 17.06 reported a VCF frequency of 14% at 24 months [37]. The current SRS literature specific to metastatic spinal sarcomas so far is reporting VCF rates lower than 10% at a median follow up time of 18 months (IQR: 12.7–31.3) in our pooled analysis [1, 4, 11, 25, 26]. Finally, there were no reported cases of radiation induced myelitis.

### Limitations and future directions

SRS has the potential to achieve good LC by achieving high biologically equivalent doses in patients with metastatic spinal sarcomas. More importantly LC in spinal sarcoma SRS does not come at the cost of increased treatment toxicity or excessive vertebral compression fracture rates relative to conventional radiation [1, 4, 11, 25, 26]. Because of this, consideration of this treatment at any point in management should be emphasized. This is particularly useful given the limitation on surgical management and the barriers to delivering effective conventional radiotherapy doses safely. Additionally, the low rates of toxicity relative to conventional radiotherapy support its role when radiotherapeutic modalities are indicated. While it is not entirely clear what the role of sarcoma histology is in influencing SRS efficacy given mixed results, it is likely that some consideration for this should be given. Furthermore, the exact dosing parameters and fractionation scheme is not necessarily apparent. In this review median doses ranged from 24 Gy to 33 Gy across 1–5 fractions, producing similar LC rate across all studies. Some of the authors in our review did report a trend toward increased LC with single versus multifraction regimens [11]. Finally, toxicity rates reported across all studies are acceptably low. The current literature is limited given the nature of retrospective studies, small sample sizes inadequate to detect the full range of effect sizes for all tumor or patient to SRS interactions. Additionally, many of the provocative questions raised by this literature such as single versus multifraction SRS, optimal dosing, the role of histological radio-resistance, the appropriate place in management for SRS will necessitate additional and larger studies. Finally, most literature reports data on both primary and metastatic lesions challenging the extraction of specific data for each subgroup. The biological behavior of these two types of tumors is most likely significantly different as to influence response to therapy. Therefore, obtaining a true estimate of the effects of SRS on these tumor subgroups requires separate analyses. Ultimately the goals of this work are to highlight the existing literature and provide an overall view of the current literature. Herein we sought to provide a framework for future research groups to reference in the design and execution of thier work. Standardized data reporting and the recruitment of more eligible cases will be paramount to enhancing the existing literature.

## Conclusion

Overall, the results of our review highlight the strengths and limitations of the existing literature in the role of SRS in metastatic sarcomas of the spine. It appears that acceptable LC at 1 year as well as OS at 1 year can be achieved by SRS in a variety of treatment settings. However, there is a steady rate of decline at 2 and 3 years respectively for both LC and OS. The exact details of how to best apply SRS in this tumor subgroup and achieve more durable responses require further investigation.

## Electronic supplementary material

Below is the link to the electronic supplementary material.

Supplementary Material 1

## Data Availability

No datasets were generated or analysed during the current study.
