# Supplementary material for: A systematic review of stereotactic radiosurgery for metastatic spinal sarcomas
Source: J Neurooncol. 2024 Nov 28;172(1):153–62. doi: 10.1007/s11060-024-04892-z (PMC11832559; doi:10.1007/s11060-024-04892-z)
Supplement: Supplementary file 1 — Supplementary Material 1 [file 11060_2024_4892_MOESM1_ESM.docx]

**A Systematic Review of Stereotactic Radiosurgery for Metastatic Spinal Sarcomas**

Trent Kite BS,^1^ Stephen Jaffe MD,^1^ Vineetha Yadlapalli BS,^2^ Rhea Verma BS,^2^ Jenna Li MS,^4^ Stephen Karlovits MD,^3^ Rodney E. Wegner MD,^3^ Matthew J. Shepard MD^1^

| **Supplementary table 1**. Lesion Characteristics | | | |
| --- | --- | --- | --- |
| **Study** | **Most common Histology** | **Anatomical Distribution (N, %)** | **Extraspinal Disease Present at Time of SRS** |
| Shanker et al [26] | Osteosarcoma | Cervical (7, 7.0%)  Thoracic (48, 48.0%)  Lumbar (27, 27.0%)  Sacral (14, 14.0%)  Thoracolumbar (3, 3.0%)  Lumbosacral (1, 1.0%) | 35% |
| Kim et al  [1] | Osteosarcoma | NR | NR |
| Bishop et al  [4] | Leiomyosarcoma | Cervical (10, 15.2%)  Thoracic (40, 60.6%)  Lumbosacral (16, 24.2%) | NR |
| Folkert et al  [11] | Leiomyosarcoma | Cervical (21, 17.5%)  Cervicothoracic (6, 5%)  Thoracic (51, 42.5%)  Thoracolumbar (3, 2.5%)  Lumbar (27, 22.5%  Lumbosacral (1,0.8%)  Sacral (11, 9.2%) | 72% |
| Levine et al  [25] | Leiomyosarcoma | NR | NR |
| Abbreviations: NR: not reported  Data reported as number of lesions unless otherwise indicated | | | |

**Supplementary table 1.** Specific lesion characteristics included in the studies included in the review.
